# Novel PVA/MOF Nanofibres: Fabrication, Evaluation and Adsorption of Lead Ions from Aqueous Solution

**DOI:** 10.1186/s11671-016-1631-2

**Published:** 2016-09-20

**Authors:** Ntaote David Shooto, Charity Wokwu Dikio, Donbebe Wankasi, Lucky Mashudu Sikhwivhilu, Fanyana Moses Mtunzi, Ezekiel Dixon Dikio

**Affiliations:** 1 Applied Chemistry and Nano-Science Laboratory, Department of Chemistry, Vaal University of Technology, P.O. Box X021, Vanderbijlpark, 1900 South Africa; 2 Advanced Materials Division, Mintek, Nanotechnology Innovation Centre, Private Bag X3015, Randburg, 2125 South Africa

**Keywords:** Electrospinning, Nanofibres, Lead, Adsorption

## Abstract

Plain polyvinyl alcohol (PVA) nanofibres and novel polyvinyl alcohol benzene tetracarboxylate nanofibres incorporated with strontium, lanthanum and antimony ((PVA/Sr-TBC), (PVA/La-TBC) and (PVA/Sb-TBC)), respectively, where TBC is benzene 1,2,4,5-tetracarboxylate adsorbents, were fabricated by electrospinning. The as-prepared electrospun nanofibres were characterized by scanning electron microscope (SEM), Fourier transform infrared (FTIR) and thermogravimetric analysis (TGA). Only plain PVA nanofibres followed the Freundlich isotherm with a correlation coefficient of 0.9814, while novel nanofibres (PVA/Sb-TBC, PVA/Sr-TBC and PVA/La-TBC) followed the Langmuir isotherm with correlation coefficients of 0.9999, 0.9994 and 0.9947, respectively. The sorption process of all nanofibres followed a pseudo second-order kinetic model. Adsorption capacity of novel nanofibres was twofold and more compared to that of plain PVA nanofibres. The thermodynamic studies: apparent enthalpy (Δ*H*°) and entropy (Δ*S*°), showed that the adsorption of Pb(II) onto nanofibres was spontaneous and exothermic. The novel nanofibres exhibited higher potential removal of Pb(II) ions than plain PVA nanofibres. Ubiquitous cations adsorption test was also investigated and studied.

**Electronic supplementary material:**

The online version of this article (doi:10.1186/s11671-016-1631-2) contains supplementary material, which is available to authorized users.

## Background

Lead is classified as one of the most poisonous elements to human health [1]. It is found in our living environment due to improper disposal. It is reported that lead is present in the air, water, soil, animal flesh and in vegetables [2]. Previously conducted studies showed that lead can cause brain damage, kidney and liver disorders and bone damages [3, 4]. After a time of accumulation in the human body, it not only jeopardises health, but also it is said to cause death even in low concentrations due to its toxic nature [5]. It is mainly used in industries such as metal plating and finishing, printing, photographic materials, explosive manufacturing, ceramic and glass manufacturing [6]. The removal of heavy metals from aqueous solution has been investigated using many nanomaterials, such as graphene [7] or graphene oxide [8], carbon nanotubes [9], polymers [10, 11] and metal-organic frameworks [12–14]; all these composites showed great potential by their high metal removal efficiency due to high surface areas. Still, many other potential adsorbents are being developed like urchin-like rutile titania carbon nanocomposites [15], sandwich-like MXene/magnetic iron oxide nanocomposites [16], and cation adsorbent [17]. Many kinds of functional materials like polymers and nanoparticles can be incorporated for achieving properties corresponding to each type of materials and integrated functionalities [18]. Some research teams have reported nanofibre hybrids, novel mesoporous polyvinyl alcohol (PVA)/SiO_2_ composite nanofibres [19] and Al(NO_3_)_3_/polyacrylonitrile (PAN) hollow nanofibres [20] intended to increase the removal efficiency of heavy metal ions from waste water.

Nanofibres are defined as long uninterrupted fibres with a diameter of 100 nm and less [21]. As the diameter of polymeric fibres decreases from micro to nano, several desirable features are exhibited, such as high surface area, unusual strength, high surface reactivity, high thermal and electric conductivity and superior mechanical properties [22–25]. Electrospinning is one of the highly favoured techniques to fabricate nanofibres due to its capabilities to produce nanofibres with controllable porous structure, versatility, ease to operate and calibre to align structures and control fibre diameters which cannot be accomplished using other conventional spinning methods [26]. It uses electrostatic forces to align electrical charges in a polymer solution and/or kinds of naturally occurring proteins, such as collagen, gelatin and silk fibroin, which have been used because of their natural abundant resources, which on drying by means of vaporization of the solvent produce nanofibres [27]. Electrospun nanofibres were proven to be useful for many applications like filtration, nanocatalysis, tissue scaffolds, protective clothing, optical electronics, biomedical, pharmaceutical and health care [28–31]. Lately, it has been found out that interactions between polymer-based gelators and nanoparticles could be employed to assemble tunable and self-healing polymer materials, where nanoparticles play an essential role in tuning the mechanical properties [32].

In this study, we have fabricated new hybrids of PVA nanofibres incorporated with Sr-TBC, La-TBC and Sb-TBC by electrospinning. These new composites were characterized and evaluated for their sorption ability to remove lead ions (Pb(II)) in aqueous solution and competing divalent ions. The results obtained are presented herein.

## Methods

### Materials

The materials are as follows: polyvinyl alcohol [(CH_2_CH(OH))_n_, PVA, fully hydrolysed; Sigma-Aldrich]; *N*,*N*-dimethylformamide [HCON(CH_3_)_2_, dimethylformamide (DMF), 99.8 %; AnalaR]; antimony potassium tartrate [K_2_Sb_2_(C_4_H_2_O_6_)_2_.3H_2_O, 99 %; Sigma-Aldrich]; strontium(II) nitrate [Sr(NO_3_)_2_, 99 %; Sigma-Aldrich]; lanthanum(II) nitrate hexahydrate [La(NO_3_)_3_.6H_2_O, 99.9 %; Sigma-Aldrich]; 1,2,4,5-tetrabenzenecarboxylic acid [C_6_H_2_(CO_2_H)_4_, 96 %; Sigma-Aldrich]; and methanol [CH_3_OH, 99.9 %; Sigma-Aldrich].

All reagents were obtained from commercial sources and were used without further purification.

### Sample Preparation

#### Preparation of Sb-TBC

The Sb-TBC was synthesized by solvothermal method. Eighty-millilitre DMF was transferred into a round bottom flask. Subsequently, K_2_Sb_2_(C_4_H_2_O_6_)_2_·3H_2_O (0.012 mol) and 1,2,4,5-tetrabenzenecarboxylic acid (0.012 mol) were dissolved in DMF by mild stirring. The solution was refluxed for 2 h at 120 °C while stirring. White crystals were obtained and isolated by centrifuge and washed with methanol to remove excess DMF, the obtained crystals were oven dried at 40 °C for 30 min and used for further experiments.

### Preparation of La-TBC

The La-TBC was synthesized by solvothermal method. Eighty-millilitre DMF was transferred into a round bottom flask. Subsequently, (0.012 mol) La(NO_3_)_3_·6H_2_O and (0.012 mol) 1,2,4,5-tetrabenzenecarboxylic acid were dissolved in DMF by mild stirring. The solution was refluxed for 2 h at 120 °C while stirring. White crystals were obtained and isolated by centrifuge and washed with methanol to remove excess DMF. The obtained crystals were oven dried at 40 °C for 30 min and used for further experiments.

### Preparation of Sr-TBC

The Sr-TBC was synthesized by solvothermal method. Eighty-millilitre DMF was transferred into a round bottom flask. Subsequently, Sr(NO_3_)_2_ (0.012 mol) and 1,2,4,5-tetrabenzenecarboxylic acid (0.012 mol) were dissolved in DMF by mild stirring. The solution was refluxed for 2 h at 120 °C while stirring. White crystals were obtained and isolated by centrifuge and washed with methanol to remove excess DMF. The obtained crystals were oven dried at 40 °C for 30 min and used for further experiments.

### Preparation for Electrospinning

The PVA solution was prepared by dissolving 2.7 g PVA into hot distilled water. The polymer solution was mixed every time with a different complex. The experimental setup used for conducting electrospinning is described previously [11].

### Lead Solution Preparation

Pb(II) stock solution (100 ppm) was prepared by dissolving 0.1 g Pb(NO_3_)_2_ in 1 L of ultrapure water. Dilutions were made to 80, 60, 40 and 20 ppm, respectively.

### Adsorption Procedure (Batch Adsorption)

#### Concentration Effect

0.1 g of nanocomposite polyvinyl alcohol incorporated with strontium benzene tetracarboxylate (PVA/Sr-TBC) nanofibre mat was weighed and placed into each of the five test tubes. Twenty millilitres of metal ion solution with standard concentration of 20, 40, 60, 80 and 100 ppm from Pb(NO_3_)_2_ solutions was transferred to each beaker containing the weighed nanocomposite. It was agitated on a shaker for 30 min; the remaining nanofibres suspension was removed by centrifugation and decanted. The remaining solutions were stored for Pb(II) analysis and ubiquitous cations using atomic adsorption spectrometer. (Same procedure was repeated for Sb-TBC and La-TBC nanofibres).

### Time Dependence Studies

0.1 g of the nanocomposite PVA/Sr-TBC nanofibre mat was weighed and transferred into each of the four test tubes. Twenty millilitres of the metal ion solution with a standard concentration of 60 ppm from Pb(NO_3_)_2_ solution was transferred to each beaker containing the weighed nanocomposite. It was agitated on a shaker for each time intervals of 5, 10, 30 and 60 min, respectively. The nanofibre suspension was centrifuged and decanted. The remaining solutions were stored for Pb(II) analysis and other competing cations. (Same procedure was repeated for Sb-TBC and La-TBC nanofibres).

### Temperature Effect

0.1 g of the nanocomposite PVA/Sr-TBC nanofibre mat was weighed and placed in four test tubes. Twenty millilitres of the metal ion solution with a standard concentration of 60 ppm from Pb(NO_3_)_2_ was transferred to a beaker containing the weighed nanocomposite. It was agitated for 30 min at temperatures of 25, 40, 60 and 80 °C, respectively, using water bath. The solution with nanofibre suspension was centrifuged and decanted. The remaining solutions were stored for Pb(II) analysis and other competing cations. (Same procedure was repeated for Sb-TBC and La-TBC nanofibres).

### Ca(II) and Mg(II) Determination

Lead solutions 20 to 100 ppm before adsorption were carried; the solutions were determined to be calcium ions (Ca(II)) and magnesium ions (Mg(II)) on atomic absorption spectroscopy (AAS). After adsorption was carried out, the same lead solutions have determined how much Ca(II) and Mg(II) ions remained in the solutions.

### Characterization

The chemical features of the as-prepared nanofibre composites were examined by scanning electron microscope (SEM), Fourier transform infrared (FTIR) and thermogravimetric analysis (TGA). The surface morphology measurements were recorded with a JOEL 7500F emission scanning electron microscope. TGA Perkin Elmer TGA 4000 was used; analyses were performed from 30 to 900 °C at a heating rate of 10 °C/min under a nitrogen atmosphere. FTIR Perkin Elmer FT-IR/FT-NIR spectrometer spectrum 400 was used. The measuring range extended from 4000 to 520 cm^−1^. After adsorption, AAS Shimadzu ASC 7000 autosampler was used to measure the remaining Pb(II) ions in the solution.

### Data Analysis

The sorbed amount of lead ions onto the adsorbent was determined using the following equation for batch dynamic studies:

1 $$ {q}_{\mathrm{e}} = \frac{V}{m}\left({C}_{\mathrm{O}}-{C}_{\mathrm{e}}\right) $$

- *q*_e_: Pb(II) concentration sorbed onto the nanocomposite at equilibria point (mg of metal ion/g of adsorbent)
- *C*_o_: Initial concentration of Pb(II) in solution (in ppm)
- *C*_e_: Equilibria point concentration of Pb(II) in solution (in ppm)
- *V*: Initial volume of Pb(II) solution used (in L)
- *m*: Weight of the nanocomposite

Langmuir graphs were plotted by applying the following equation:

2 $$ \frac{m}{x} = \frac{1}{ab{C}_e} + \frac{1}{b} $$

- *x*: Pb(II) sorbed per mass of nanocomposite (in mg/L)
- *a* and *b*: The Langmuir constants obtained from the slope and intercepts of the plots

The Langmuir isotherm was showed in terms of an equation of separation factor *S*_*f*_. It determines a type of adsorption isotherm. When *S*_*f*_ is greater than 1, the isotherm is unfavourable; if *S*_*f*_ is 1, linear, if 0  <  *S*_*f*_  <  1.0, favourable; and *S*_*f*_  =  0, irreversible.

3 $$ {S}_f = \frac{1}{1 + a{C}_o} $$

The degree of surface coverage of adsorbent covered by lead ions was calculated using

4 $$ \theta =1 - \frac{C_{\mathrm{e}}}{C_o} $$

The capability of nanofibres to reduce the amount of Pb(II) in solution was evaluated by total cycles of equilibrium adsorption needed according to the value of the partition coefficient (*K*_d_) in Eq. 5

5 $$ {K}_{\mathrm{d}} = \frac{C_{\mathrm{ads}}}{C_{\mathrm{aq}}}, $$

where

- *C*_aq_: Concentration of Pb(II) in solution (in mg/L)
- *C*_ads_: Concentration of Pb(II) in nanocomposite (in mg/L)

The Suzuki equation was used to determine the heat of adsorption (*Q*_ads_) as expressed in the below equation:

6 $$ \mathrm{In}\ \theta = \frac{\mathrm{In}{K}_{\mathrm{O}}{C}_{\mathrm{O}}}{T^{0.5}} + \frac{Q_{\mathrm{ads}}}{RT}, $$

where

- *T*: Temperature of the solution (in K)
- *K*_0_: Constant
- *R:* Gas constant (8.314 J/Kmol)

The linearized Arrhenius equation was used to the obtained data to determine the activation energy (*E*_a_) and sticking probability S*

7 $$ \mathrm{In}\left(1 - \theta \right)={S}^{*} + \frac{E_{\mathrm{a}}}{RT} $$

Gibbs free energy (Δ*G*^o^) of the sorption process was applied to evaluate the spontaneity.

8 $$ \varDelta {G}^{{}^{\circ}}=RT\mathrm{In}{K}_{\mathrm{d}} $$

Further investigation was done to measure the enthalpy (Δ*H*°) and entropy (Δ*S*°) of the sorption process by using Eq. 9.

9 $$ \varDelta {G}^{{}^{\circ}} = \varDelta {H}^{{}^{\circ}} - T\varDelta {S}^{{}^{\circ}} $$

The number of hopping (*n*) was calculated by relating it to the surface coverage (*θ*).

10 $$ n = \frac{1}{\left(1-\theta \right)\theta } $$

## Results and Discussion

**Fig. 1:**
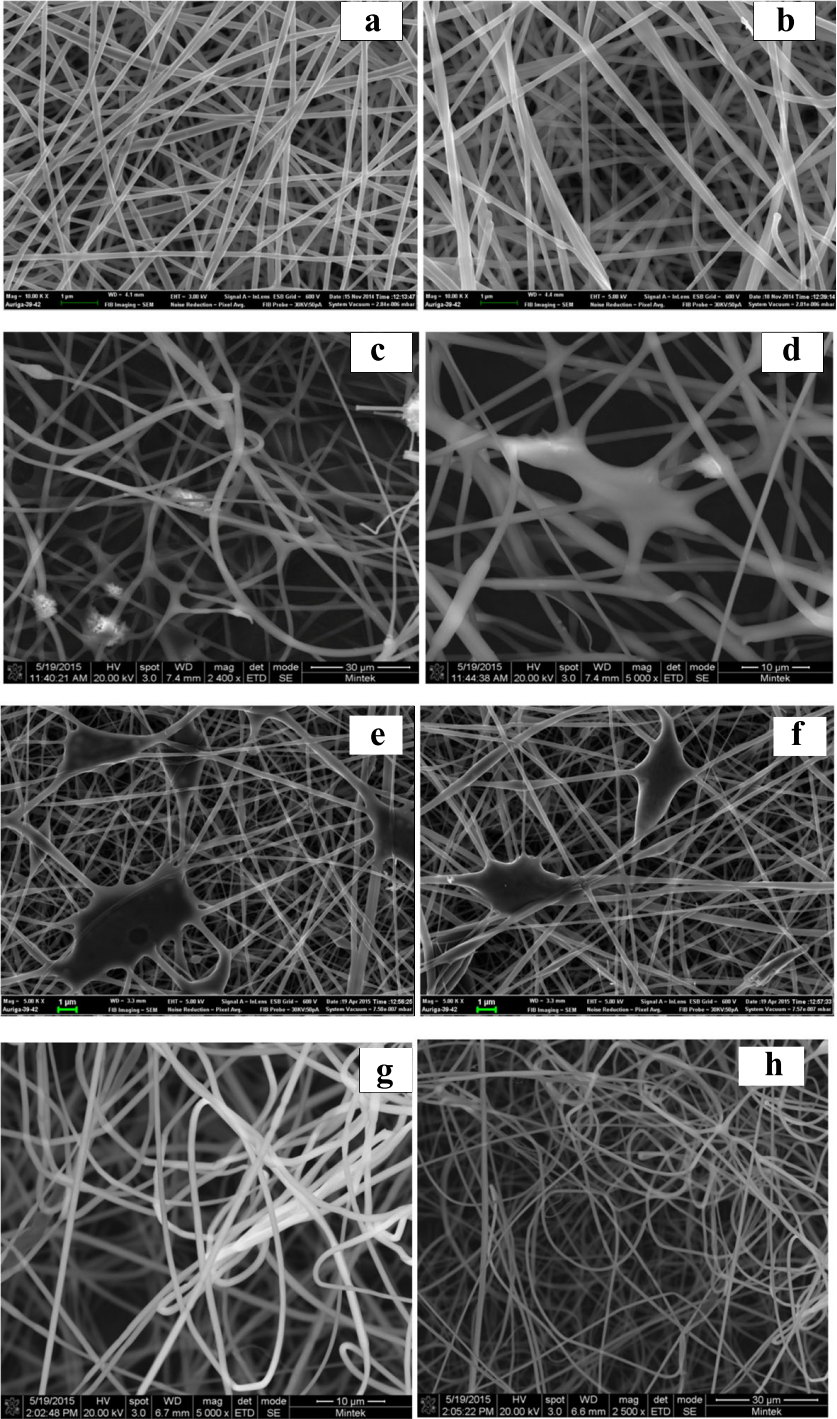
**a**, **b** SEM micrographs of PVA nanofibres. **c**, **d** Micrographs of PVA/Sr-TBC nanofibres. **e**, **f** Micrographs of PVA/La-TBC nanofibres. **g**, **h** Micrographs of PVA/Sb-TBC nanofibres

The SEM micrographs of electrospun PVA nanofibres composites are presented in Fig. 1a–h. Plain PVA nanofibres are shown in Fig. 1a, b; it is observed that uniform, smooth and continuous bead free nanofibres were fabricated. The nanofibre morphology exhibited no apparent surface and structural defects. PVA/Sr-TBC (Fig. 1c, d) and polyvinyl alcohol incorporated with lanthanum benzene tetracarboxylate (PVA/La-TBC; Fig. 1e, f) nanofibre micrographs showed fibrous morphology having patching areas that connected multiple nanofibres forming semi-cross linkage. These patching areas are aggregates, formed to indicate molecular interaction between PVA nanofibres and Sr-TBC and La-TBC, respectively; from the observations, it is therefore concluded that mobilisation of PVA nanofibres with Sr-TBC and La-TBC was successfully executed. In Fig. 1g, h, PVA/Sb-TBC nanofibre micrographs showed much entanglement and network structures; this could be due to the presence of Sb-TBC embedded in the PVA nanofibres.

**Fig. 2:**
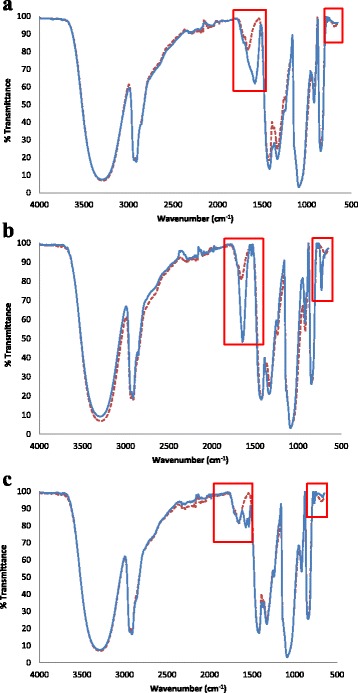
**a** FTIR spectra of PVA nanofibres (*red dotted line*) vs. PVA/La-TBC nanofibres (*blue solid line*). **b** PVA nanofibres (*red dotted line*) vs. PVA/Sb-TBC nanofibres (*blue solid line*) and **c** PVA nanofibres (*red dotted line*) vs. PVA/Sr-TBC nanofibres (*blue solid line*)

Plain PVA nanofibres were used as control in order to monitor the changes that occurred on the FTIR peaks of the nanofibres incorporated with complexes. FTIR spectra of the investigated composites are similar as shown in Fig. 2a–c, showing that they are fabricated by the same material, PVA. Figure 2a shows the PVA vs. PVA/La-TBC composite nanofibres. On the spectra of electrospun PVA nanofibre mat, the major peaks observed were as follows: bands at 3293.85 and 1660.20 cm^−1^ are assigned to the stretching and bending vibrations of (O–H), respectively; two vibration bands at 2936.50 and 2911.11 cm^−1^ are assigned to (C–H); a sharp band at 1100.27 is attributed to the vibration of (C–C); and a medium shoulder peak at 917.30 cm^−1^ is linked to the vibration of (C–O). In the spectrum of PVA/La-TBC nanofibres, two major changes were observed a medium peak at 1581.11 cm^−1^ and a newly formed peak at 690.99 cm^−1^ as highlighted on the spectra. In Fig. 2b, PVA/Sb-TBC nanofibre IR spectra also showed multiple major changes, a sharp peak at 1644.73 cm^−1^ and two new peaks: one at 1539.84 could not be clearly seen owing to the apparent overlapping and other at 727 cm^−1^. In Fig. 2c, PVA/Sr-TBC nanofibres newly formed multiple peaks were observed 1572, 1539 and 767 cm^−1^. Therefore, it is concluded that there were a given amount of complexes embedded in the electrospun fibrous mat.

**Fig. 3:**
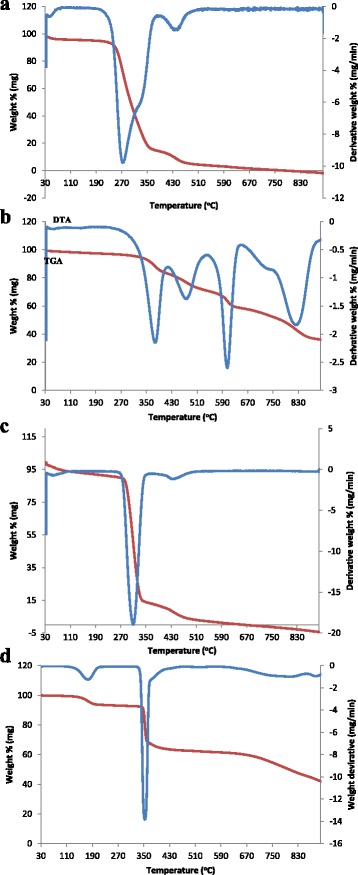
Thermogravimetric analysis (TGA) and derivative thermogravimetric analysis (DTA) of **a** PVA nanofibres, **b** PVA/Sr-TBC nanofibres, **c** PVA/La-TBC nanofibres, and **d** PVA/Sb-TBC nanofibres

Thermal stability of the fabricated nanofibres adsorbents material was assessed. Figure 3a–d shows the TGA and differential thermogravimetric analyses (DTA) that were conducted on the fabricated nanofibres. In Fig. 3a, plain PVA nanofibre plot showed three main decomposition steps: the first decomposition occurred between 30 and 75 °C which was mainly due to the dehydration of water molecules within the fibres that was physisorbed [33]. The second decomposition occurred between 149 and 371 °C of which this was the most intense weight loss corresponding to the side chains of PVA, the loss of H-bond between PVA molecules and O-bond between C–O. The third decomposition 378–489 °C corresponds to the disintegration of the main chain of PVA [34]. TGA and DTA plots demonstrated that PVA nanofibres are highly unstable at high temperatures especially from 508 °C as no residue remained after the analysis. In Fig. 3b, d, the PVA/Sr-TBC and PVA/Sb-TBC nanofibres were more thermally stable and exhibited higher decomposition points than plain PVA as the remaining residue was 36.96 and 36.87 %, respectively, at the end of the run. Figure 3c was thermally unstable as plain PVA nanofibres as no residue remained after the thermal assessment test.

**Fig. 4:**
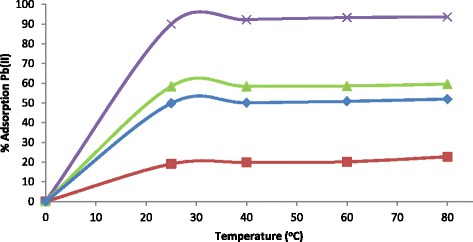
Temperature effect on the sorption of Pb(II) ions onto PVA nanofibres (*filled square*), PVA/Sb-TBC nanofibres (*filled diamond*), PVA/Sr- TBC nanofibres (*filled triangle*) and PVA/La-TBC nanofibres (*X mark*)

To better understand the significant role of temperature on Pb(II) ion sorption on the adsorbents, temperature effect experiments were conducted at four different temperatures (25, 40, 60 and 80 °C) as shown in Fig. 4. It was observed in all instances that the sorption rate was very rapid from 0 to 25 °C, but as temperature increased, sorption rate was slowed. Sorption slightly increased though negligible and cannot be clearly seen on the plots; at temperatures 25, 40, 60 and 80 °C, percent adsorption was PVA = 19.0, 19.8, 20.1 and 22.7; PVA/Sb-TBC = 49.8, 50.1, 50.8 and 51.9; PVA/Sr-TBC = 58.1, 58.4, 58.6 and 59.6; and PVA/La-TBC = 90.0, 92.2, 93.3 and 93.3, respectively. These results suggests that temperature has no significant role on the sorption of Pb(II) onto nanofibre composites.

The activation energy (*E*_a_) and the sticking probability (S*) were calculated from Eq. 7. *E*_a_ and S* values were −2.158 and 0.7734 (PVA), −6.1532 and 0.4825 (PVA/Sb-TBC), −7.51 and 0.4068 (PVA/Sr-TBC) and −24.63 and 0.0725 (PVA/La-TBC), respectively, as shown in Tables 1 and 2. Negative activation energy (−*E*_a_) indicates the absence of energy barrier to cause the sorption to occur. The sticking probability S* measures the potential of an adsorbate to remain on the adsorbent. It is often interpreted as S* > 1 (no sorption), S* = 1 (mixture of physisorption and chemisorption), S* = 0 (indefinite sticking—chemisorption), 0 < S* < 1 (favourable sticking—physisorption).

**Table 1:** Thermodynamic parameters of the nanocomposites

	*T* (K)	Δ*G* ^o^ (KJ/mol)	Δ*H*° (KJ/mol)	Δ*S*° (J/mol)	*E*_a_ (KJ/mol)
Sorption of Pb(II) onto PVA nanofibres	298	3.45	−2.68	2.9	−2.158
	313	3.63			
	333	3.82			
	353	3.59			
Sorption of Pb(II) onto PVA/Sr-TBC nanofibres	298	−8.40	−760.54	−5.29	−7.51
	313	−8.83			
	333	−9.58			
	353	−11.37			
Sorption of Pb(II) onto PVA/La-TBC nanofibres	298	−5.45	−7348.3	−43.50	−24.63
	313	−6.41			
	333	−7.29			
	353	−7.86			
Sorption of Pb(II) onto PVA/Sb-TBC nanofibres	298	−5.45	−308.84	0.9488	−6.1532
	313	−6.41			
	333	−7.29			
	353	−7.86

**Table 2:** Thermodynamic parameters of the nanocomposites

Composite	Heat of adsorption, *Q*_ads_ (KJ/mol K)	Sticking probability (S*)	Hopping number, *n*	Adsorption potential, *A* (KJ/mol)
PVA nanofibres	−5.3145	0.7734	1	0.7566
PVA/Sb-TBC nanofibres	−10.41	0.4825	4	−2.0727
PVA/Sr-TBC nanofibres	−10.91	0.4068	4	−1.834
PVA/La-TBC nanofibres	−16.32	0.0725	11	−7.5595

Hopping number (*n*) estimates the chance of Pb(II) ions finding vacant sites on the surface of the nanofibre composites during sorption was calculated as shown in Table 2. The hopping number was 1 (PVA), 4 (PVA/Sb-TBC), 4 (PVA/Sr-TBC) and 11 (PVA/La-TBC). The lower the hopping number, the faster the sorption process. The low value of *n* obtained in this study suggests that the sorption of Pb(II) on nanofibre composites was very fast.

**Fig. 5:**
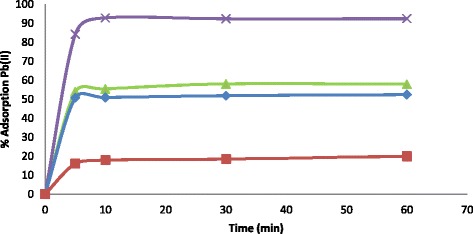
Time dependence studies on the sorption of Pb(II) ions onto PVA nanofibres (*filled square*), PVA/Sb-TBC nanofibres (*filled diamond*), PVA/Sr-TBC nanofibres (*filled triangle*) and PVA/La-TBC nanofibres (*X mark*)

Another important parameter to understand is the effect of time on Pb(II) ion sorption at time intervals (5, 10, 30 and 60 min). Figure 5 shows the time required for maximum sorption to occur. It was observed in all instances that Pb(II) sorption was very fast especially at the starting period. This was because of the readily accessible vacant sites at the initial stage of the sorption process but as the sorption sites decreased in number and became exhausted, the sorption rate was slowed down. The maximum percentage sorption of Pb(II) ions was as follows: PVA nanofibres increased from 16.1 % in 5 min to 20 % in 60 min; PVA/Sb-TBC nanofibres increased from 50.7 % in 5 min to 52.7 in 60 min; PVA/Sr-TBC nanofibres increased from 53.8 % in 5 min to 57.9 in 60 min; and PVA/La-TBC nanofibres increased from 84.2 % in 5 min to 92.4 in 60 min.

**Fig. 6:**
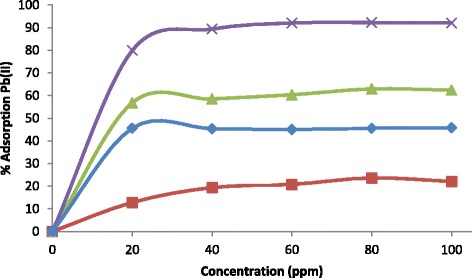
Concentration effect on the sorption of Pb(II) ions onto PVA nanofibres (*filled square*), PVA/Sb-TBC nanofibres (*filled diamond*), PVA/Sr-TBC nanofibres (*filled triangle*) and PVA/La-TBC nanofibres (*X mark*)

To evaluate the sorption capability of Pb(II) ions on PVA nanocomposites at different Pb(II) concentrations (20, 40, 60, 80 and 100 ppm), Fig. 6, the sorption rate was noticeably very rapid at lower concentrations particularly on the 20- and 40-ppm solutions; this was due to the presence of large number of vacant bounding sites and free pore space on the nanofibre surface, but as concentration further increased, sorption capability slowed down especially from the 60-ppm solution, indicating the saturation of vacant sites and pores. From that point, no further sorption was observed; this showed equilibrium was reached.

**Fig. 7:**
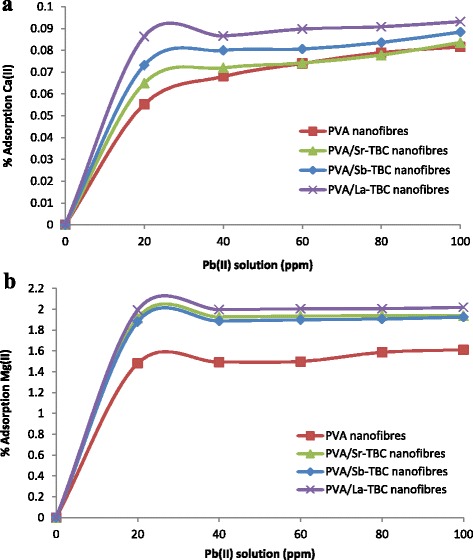
**a** Uptake of trace amount of Ca(II) ions in lead ion solution. **b** Uptake of trace amount of Mg(II) ions in lead ion solution

**Scheme 1:**
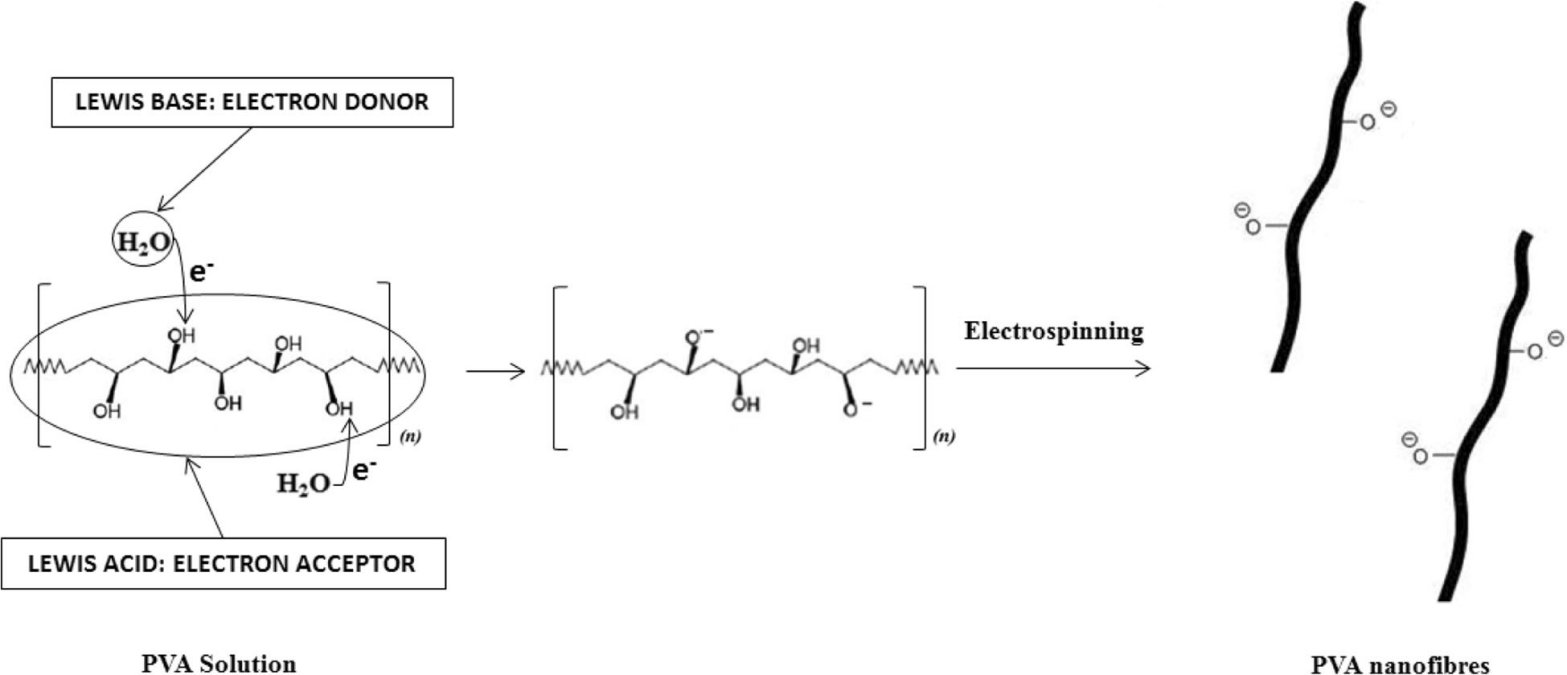
Proposed PVA nanofibre formation mechanism and functional groups present in the nanofibres

**Scheme 2:**
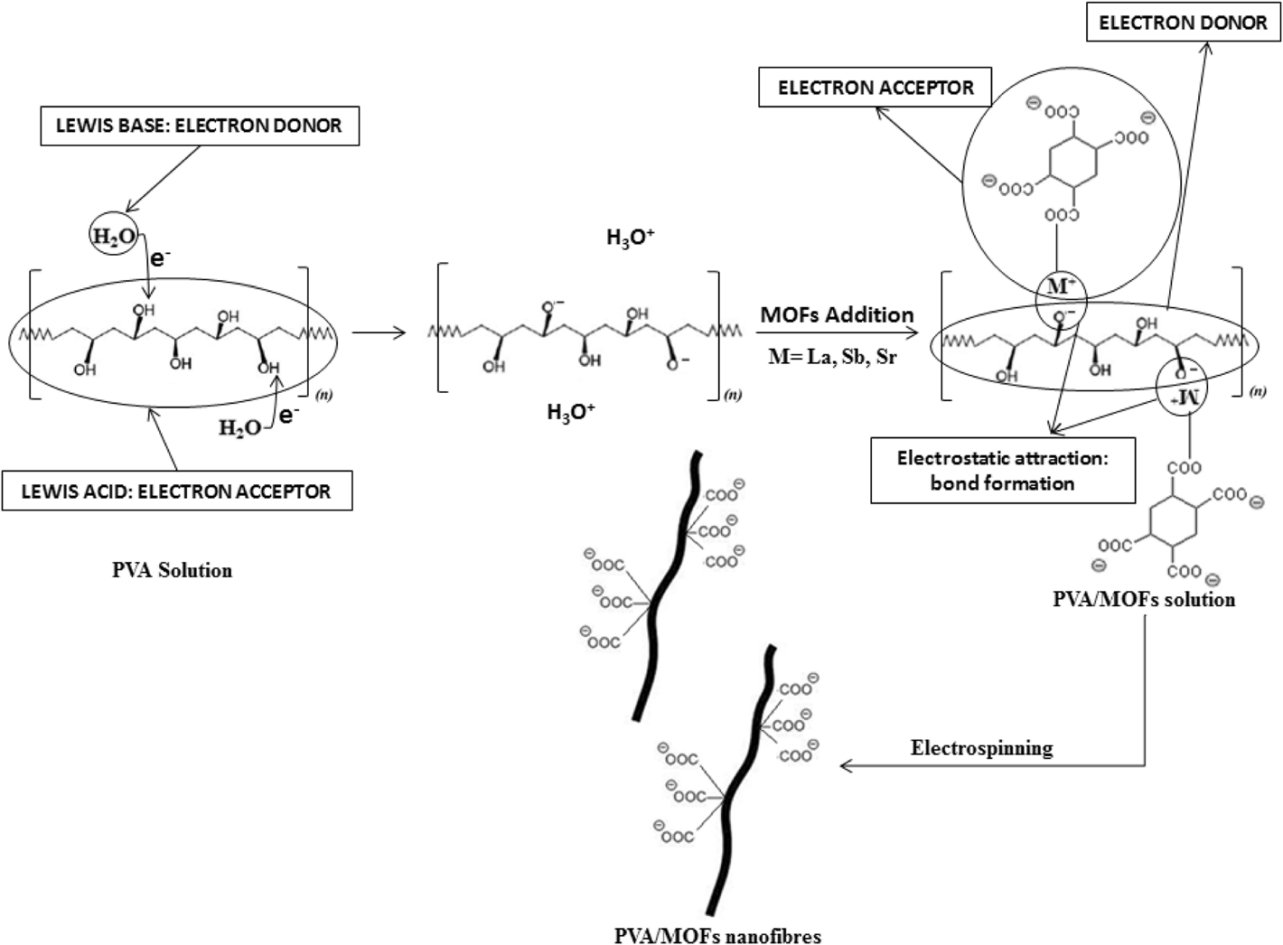
Proposed PVA/MOF nanofibre formation mechanism and functional groups present in the nanofibres

It is known that water contains ubiquitous cations such as Ca(II) and Mg(II); these divalent ions have the same positive charge as Pb(II), and this leads to serious adsorption competition towards heavy metal removal [35]. Therefore, it is essential to evaluate the adsorption performance of PVA and PVA/MOFs nanofibres for Pb(II) and competing cations. Adsorptions of these ions in the test solution were studied in ultrapure water. The prepared lead(II) stock solution with possible Ca(II) and Mg(II) ions was used for adsorption of Pb(II) and ubiquitous ions in the study. Measurements were carried out on the prepared solutions for calcium and magnesium ion adsorption in the presence of lead ions before lead adsorption studies and after adsorption of lead studies. Figure 7a, b shows the adsorption of these divalent metal ions in the Pb(II) ion solution. The percentage adsorption of magnesium and calcium ions adsorbed by the PVA/MOF nanofibres is low. This shows that there was not a high degree of competition from these ions in the water source used in the study. Mg(II) ions exhibited higher adsorption due to its more prominent electronegativity (1.31, Pauling scale) and smaller atomic radius (145 pm) than Ca(II) which has lesser electronegativity (1.00, Pauling scale) and bigger atomic radius (194 pm). Higher uptake was exhibited in PVA/MOFs than plain PVA nanofibres. This is due to abundant carboxylate groups compared to hydroxyl groups in plain PVA nanofibres, as depicted in Schemes 1 and 2. PVA/MOF nanofibres exhibited greater liberation of Pb(II) and competing ions than plain PVA nanofibres; this confirmed the advantage of incorporated nanofibres than the plain nanofibres.

Schemes 1 and 2 depict the PVA and PVA/MOF nanofibre formation, where PVA acts as the Lewis acid (electron acceptor) in water medium (Lewis base) electron donor. Oxygen in water molecule donated its unbound electron to cleave hydrogen from hydroxyl groups (O–H) on PVA molecules. In Scheme 2, after MOFs are added, the same process is repeated where negatively charged oxygen on PVA and positively charged metal bound to organic framework are electrostatically attracted and share oxygen-free unbound electrons to form a bond.

Sorption distribution (*K*_d_) was used to evaluate the performance of nanofibres as potential adsorbents Pb(II) from solution is presented in Table 3. The values of *K*_d_ (3.5315 (PVA), 1.1993 (PVA/Sb-TBC), 0.6634 (PVA/Sr-TBC) and 0.2264 (PVA/La-TBC)) suggest that nanofibres are efficient adsorbents; however, more cycles of equilibrium sorption process are needed to reduce the levels of Pb(II) in the solution.

**Table 3:** Equilibrium and kinetic parameters

Composite	Surface coverage (*θ*)	Separation factor (*S*_*f*_)	Sorption coefficient (*K*_d_)	Percentage adsorption (mol/g)
PVA nanofibres	0.2206	0.7028	3.5315	25.50
PVA/Sb-TBC nanofibres	0.5064	0.3981	1.1993	50.66
PVA/Sr-TBC nanofibres	0.5874	0.3577	0.6634	58.85
PVA/La-TBC nanofibres	0.9227	0.3508	0.2264	92.27

Separation factor (*S*_*f*_) was applied to determine the nature of the sorption process, whether Pb(II) sorption unto nanofibres was favourable or not. The *S*_*f*_ values were 0.7028 (PVA), 0.3981 (PVA/Sb-TBC), 0.3577 (PVA/Sr-TBC) and 0.3508 (PVA/La-TBC) as presented in Table 3. All *S*_*f*_ values were below 1 and more than 0; this indicates that the sorption of Pb(II) ions was favourable.

Surface coverage (*θ*) is given in Table 3 as 0.2206 = 22.06 % (PVA), 0.5064 = 50.64 (PVA/Sb-TBC), 0.5874 = 58.74 (PVA/Sr-TBC) and 0.9227 = 92.27 % (PVA/La-TBC). These values indicated that high percentage of the active sites of nanofibre surface were covered by Pb(II), which means that the highest degree of sorption occurred in PVA/La-TBC; thus, it has higher surface coverage and adsorption capacity as shown in Table 3, followed by PVA/Sr-TBC, PVA/Sb-TBC and plain PVA nanofibres.

Percentage adsorption of the produced novel nanofibres was 92.27 (PVA/La-TBC), 58.85 (PVA/Sr-TBC) and 50.66 (PVA/Sb-TBC).

Heat of adsorption (*Q*_ads_) for the sorption of Pb(II) ions was calculated and obtained the values of −5.3145 (PVA), −10.41 (PVA/Sb-TBC), −10.91 (PVA/Sr-TBC) and −16.32 (PVA/La-TBC) as presented in Table 2. Negative values indicated that the sorption was exothermic. Pb(II) sorbed onto the electrospun nanofibres favoured low temperatures. Thus, increased temperatures did not improve the sorption processes.

The Gibbs free energy (Δ*G*^o^) was calculated from Eq. 8 as presented in Table 1. Δ*G*^o^ aids to determine the spontaneity of the sorption process. The calculated Δ*G*^o^ values for PVA/Sb-TBC, PVA/Sr-TBC and PVA/La-TBC nanofibres were negative indicating that the sorption was spontaneous; no external energy was required to initiate the sorption process. Only plain PVA nanofibre Δ*G*^o^ values were positive.

The apparent enthalpy (Δ*H*°) and entropy (Δ*S*°) of the sorption calculated from Eq. 9 values are shown in Table 1. The values of enthalpy change (Δ*H*°) and entropy (Δ*S*°), respectively, were −2.68 and 2.9 (PVA), −308.84 and 0.9488 (PVA/Sb-TBC), −760.54 and −5.29 (PVA/Sr-TBC), and −7348.3 and −43.50 (PVA/La-TBC). Negative values for Δ*H*° suggested that the sorption favoured lower temperatures. The entropy change Δ*S*° gave positive values for PVA and PVA/Sb-TBC nanofibres; this means that Pb(II) ions were not restricted in the electrospun nanofibres, and physisorption mechanism was dominant. Chemisorption was more dominant in the sorption of PVA/La-TBC and PVA/Sr-TBC nanofibres as the obtained Δ*S*° values were negative.

Additional file 1: Figures S1 and S2 (Supporting information) is the plots of the Langmuir and Freundlich isotherms and their corresponding correlation coefficients (*R*^2^) are given in Table 4. Sorption data of PVA/Sb-TBC, PVA/Sr-TBC and PVA/La-TBC nanofibre (*R*^2^) magnitudes best fitted the Langmuir isotherm. Plain PVA nanofibres sorption data followed the Freundlich isotherm.

**Table 4:** Isotherm parameters of the Langmuir and Freundlich correlation coefficients (*R* ^2^)

	Correlation coefficients (*R* ^2^)	
	Langmuir	Freundlich
PVA nanofibres	0.9734	0.9814
PVA/Sb-TBC nanofibres	0.9999	0.9997
PVA/Sr-TBC nanofibres	0.9994	0.9976
PVA/La-TBC nanofibres	0.9947	0.9249

Additional file 1: Figure S3 (Supporting information) presents the plots of the pseudo second order model. Best-fitted kinetic model is selected based upon the magnitude of the obtained correlation coefficients (*R*^2^). The magnitudes of the R^2^ for the pseudo second-order model were greater than those of other kinetic models as shown in Table 5. Therefore, it was concluded that the sorption data suited best the pseudo second-order kinetic mechanism.

**Table 5:** Kinetics parameters for Pb(II) sorption correlation coefficients (*R* ^2^)

	Correlation coefficients (*R* ^2^)
	Pseudo second order
PVA nanofibres	0.9495
PVA/La-TBC nanofibres	0.9999
PVA/Sb-TBC nanofibres	0.9910
PVA/Sr-TBC nanofibres	0.9999

**Fig. 8:**
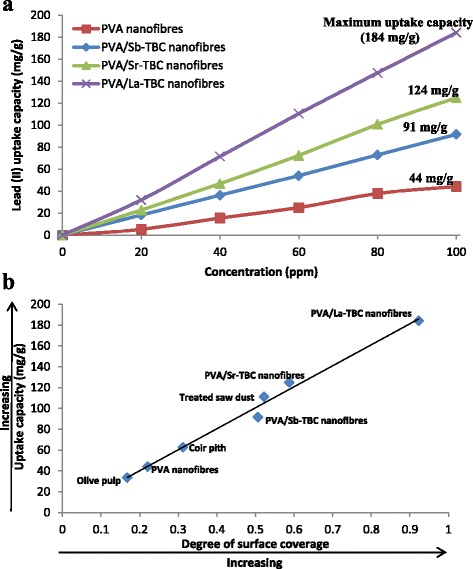
**a** Uptake capacity of PVA and novel incorporated nanofibres. **b** Maximum uptake capacity of nanofibres against some activated carbons

Common conventional adsorbents used for heavy metal removal is activated carbon which is prepared from a variety of carbon-containing materials; activated carbon prepared from coir-pith gave a maximum uptake capacity of 62.5 mg/g [36], that prepared from olive pulp has 33.6 mg/g [37] and that treated with saw dust has 111 mg/g [38]. We herein report higher performance novel PVA/Sb-TBC, PVA/Sr-TBC and PVA/La-TBC nanofibres with maximum uptake capacity of 91, 124 and 194 mg/g, respectively, as shown in Fig. 8a plots. Figure 8b provides a better understanding on how so PVA/MOFs exhibited higher uptake capacity; the figure also shows the correlation between the degree of surface coverage and uptake capacity. It was observed that as the degree of surface coverage increased so also the uptake capacity. Higher uptake capacity exhibited by MOF-enriched nanofibres than some commonly used activated carbon and plain PVA nanofibres was because more surface area was facilitated for adsorption in MOFs/PVA nanofibres. Hussain et al. [39] reported that blended or functionalized nanofibres were found to have smaller diameters with narrower diameter distributions than pure unfunctionized nanofibres. Diameters of enriched PVA nanofibres decreased as MOF content were added. This was due to the increase in conductivity of the solution as MOFs were introduced. Decreased diameters of PVA/MOF nanofibres led to higher uptake capacity. Fabricated enriched nanofibres have proven to be good candidates for this application, which demonstrated to be more effective lead ions adsorbents, exceeding some of the commonly used activated carbon. This research proposes a convenient approach for the application of incorporated nanofibres in the field of practical water treatment and the advantages of using polymeric nanofibre adsorbents. Results from this work will add to the knowledge base on the fabrication, characterization and the use of incorporated PVA nanofibres for the adsorption studies.

## Conclusions

The present investigation showed that novel PVA/La-TBC, PVA/Sr-TBC and PVA/La-TBC nanofibres were successfully fabricated by electrospinning. TGA-DTA plots and FTIR spectra confirmed PVA nanofibres and complexes incorporation. The produced nanofibres were applied as potential adsorbents for heavy metal [Pb(II)] treatment from contaminated water systems. The chemisorption predominated adsorbents (PVA/La-TBC and PVA/Sr-TBC nanofibres) removed the Pb(II) ions much better than the physisorption dominant adsorbents (plain PVA and PVA/Sb-TBC nanofibres). PVA/Sb-TBC, PVA/Sr-TBC and PVA/La-TBC nanofibre sorption data fitted best with the Langmuir isotherm, indicating the homogeneous nature of the monolayer sorption of Pb(II) on the modified nanofibres. Sorption of Pb(II) onto the nanofibres was rapid and spontaneous. This research proposes a convenient approach for the application of modified nanofibres in the field of practical water treatment.

## Additional File

Additional file 1: Figure S1. The Langmuir isotherm plots, M/X against 1/*C*_e_. (a) PVA nanofibres, (b) PVA/Sb-TBC nanofibres, (c) PVA/La-TBC nanofibres and (d) PVA/Sr-TBC nanofibres. Figure S2. The Freundlich isotherm plots, InX/M against In*C*_e_. (a) PVA nanofibres, (b) PVA/Sb-TBC nanofibres, (c) PVA/La-TBC nanofibres and (d) PVA/Sr-TBC nanofibres. Figure S3. Plots of *t*/*q*_*t*_ vs. *t* for adsorption of Pb^2+^ onto (a) PVA nanofibres, (b) PVA/Sb-TBC nanofibres, (c) PVA/La-TBC nanofibres and (d) PVA/Sr-TBC nanofibres. (DOCX 74 kb)

## Abbreviations

*a* and *b*: Langmuir constants obtained from the slope and intercepts of the plots
Ca(II): Calcium ions
*C*_ads_: Concentration of Pb(II) in nanocomposite
*C*_aq_: Concentration of Pb(II) in solution
*C*_e_: Equilibria point concentration of Pb(II) in solution
*C*_o_: Initial concentration of Pb(II) in solution
DMF: Dimethylformamide
FTIR: Fourier transform infrared
*m*: Weight of the nanocomposite
Mg(II): Magnesium ions
Pb(II): Lead ions
PVA: Polyvinyl alcohol
PVA/La-TBC: Polyvinyl alcohol incorporated with lanthanum benzene tetracarboxylate
PVA/Sb-TBC: Polyvinyl alcohol incorporated with antimony benzene tetracarboxylate
PVA/Sr-TBC: Polyvinyl alcohol incorporated with strontium benzene tetracarboxylate
*q*_e_: Pb(II) concentration adsorbed onto nanocomposite at equilibria point
*R*: Gas constant (8.314 J/Kmol)
SEM: Scanning electron microscope
TGA: Thermogravimetric analysis
*V*: Initial volume of Pb(II) solution used
*x*: Pb(II) adsorbed per mass of nanocomposite
Δ*H°*: Enthalpy
Δ*S°*: Entropy
$$ K $$: Constant
*T*: Temperature of the solution
