# Supplementary material for: Novel PVA/MOF Nanofibres: Fabrication, Evaluation and Adsorption of Lead Ions from Aqueous Solution
Source: Nanoscale Res Lett. 2016 Sep 20;11:414. doi: 10.1186/s11671-016-1631-2 (PMC5028355; doi:10.1186/s11671-016-1631-2)
Supplement: Additional file 1: Figure S1. — The Langmuir isotherm plots, M/X against 1/C e. (a) PVA nanofibres, (b) PVA/Sb-TBC nanofibres, (c) PVA/La-TBC nanofibres and (d) PVA/Sr-TBC nanofibres. Figure S2. The Freundlich isotherm plots, InX/M against InC e. (a) PVA nanofibres, (b) PVA/Sb-TBC nanofibres, (c) PVA/La-TBC nanofibres and (d) PVA/Sr-TBC nanofibres. Figure S3. Plots of t/q t vs. t for adsorption of Pb2+ onto (a) PVA nanofibres, (b) PVA/Sb-TBC nanofibres, (c) PVA/La-TBC nanofibres and (d) PVA/Sr-TBC nanofibres. (DOCX 74 kb) [file 11671_2016_1631_MOESM1_ESM.docx]

**Supporting documents**

**(a)**

**(b)**

**(c)**

**(d)**

**Fig. S1** Shows Langmuir isotherm plots, M/X against 1/C_e_ (a) PVA nanofibres and (b) PVA/Sb-TBCA nanofibres, (c) PVA/La-TBCA nanofibres and (d) PVA/Sr-TBCA nanofibres.

**(a)**

**(b)**

**(c)**

**(d)**

**Fig. S2** Shows Freundlich isotherm plots, InX/M against InC_e_ (a) PVA nanofibres and (b) PVA/Sb-TBCA nanofibres, (c) PVA/La-TBCA nanofibres and (d) PVA/Sr-TBCA nanofibres.

**(a)**

**(b)**

**(c)**

**(d)**

**Fig. S3** Plots of t/q_t_ vs. t for adsorption of Pb^2+^ onto (a) PVA nanofibres and (b) PVA/Sb-TBCA nanofibres, (c) PVA/La-TBCA nanofibres and (d) PVA/Sr-TBCA nanofibres.
